# AxSpA patients who also meet criteria for fibromyalgia: identifying distinct patient clusters using data from a UK national register (BSRBR-AS)

**DOI:** 10.1186/s41927-019-0066-7

**Published:** 2019-05-20

**Authors:** Gary J. Macfarlane, Ejaz Pathan, Stefan Siebert, Jonathan Packham, Karl Gaffney, Ernest Choy, Raj Sengupta, Fabiola Atzeni, Kathryn R. Martin, Gareth T. Jones, Linda E. Dean

**Affiliations:** 1 Epidemiology Group, School of Medicine, Medical Sciences and Nutrition and The Aberdeen Centre for Arthritis and Musculoskeletal Health, University of Aberdeen, Health Sciences Building, Foresterhill, Aberdeen, AB25 2ZD UK; 2 Department of Rheumatology, Spondylitis Program, Toronto Western Hospital, University Health Network, Toronto, Ontario Canada; 3 Institute of Infection, Immunity & Inflammation, College of Medical, Veterinary & Life Sciences, University of Glasgow, Glasgow, UK; 4 Institute of Primary Care and Health Sciences, Keele University, Keele, UK; 5 Department of Rheumatology, Norfolk & Norwich University Hospitals NHS Foundation Trust, Norwich, UK; 6 CREATE Centre, Section of Rheumatology, Division of Infection and Immunity, Cardiff University School of Medicine, Cardiff, UK; 7 Royal National Hospital for Rheumatic Diseases, Bath, UK; 8 Rheumatology Unit, L. Sacco University Hospital, Milan, Italy

**Keywords:** Axial spondyloarthritis, Fibromyalgia, Comorbidity, Criteria, Disease register, Cluster analysis

## Abstract

**Background:**

Around 1 in 8 patients with axial spondyloarthritis (axSpA) also meet criteria for fibromyalgia and such patients have considerable unmet need. Identifying effective therapy is important but to what extent fibromyalgia-like symptoms relate to axSpA disease severity has not been established. The aim of the current analysis was to determine whether distinct clusters of axSpA patients exist and if so to determine a) whether they differ in terms of prevalence of fibromyalgia and b) the features of patients in clusters with high prevalence.

**Methods:**

The British Society for Rheumatology Biologics Register (BSRBR-AS) recruited axSpA patients from 83 centres 2012–2017. Clinical data, and information from patients was collected (including research criteria for fibromyalgia). Cluster analysis was undertaken using split samples for development and validation both in the whole population and the sub-group which met fibromyalgia criteria.

**Results:**

One thousand three hundred thirty-eight participants were included of whom 23% met research criteria for fibromyalgia. Four clusters were identified. Two exhibited very high disease activity, one which was primarily axial (*n* = 347) and a smaller cluster (*n* = 32) with axial and peripheral disease, and in both groups more than half of members met criteria for fibromyalgia. The remaining two clusters (*n* = 437, *n* = 462) had overall less severe disease however the one which showed greater disease activity and poorer quality of life had a higher proportion meeting fibromyalgia criteria (16% v. 4%). Within those meeting fibromyalgia criteria there were three clusters. The two main groups were defined by level of symptom severity with a smaller third cluster noted to have high average swollen and tender joint counts and high levels of comorbidity.

**Conclusions:**

The major feature defining clusters with a high proportion of persons meeting criteria for fibromyalgia is high axSpA disease activity although clusters with features of fibromyalgia in the absence of high disease activity also show moderately high prevalence. Management may be most successful with pharmacologic therapy to target inflammation but enhanced by the concurrent use of non-pharmacologic therapy in such patients.

## Background

Fibromyalgia is common as a co-morbidity in inflammatory arthritis. A recent meta-analysis estimated the prevalence as 21% (95% CI 17, 25) in rheumatoid arthritis (RA) across 25 studies, 13% (95% CI 7, 19) in axial spondyloarthritis (axSpA) across eight studies and 18% (95% CI 13, 23) in psoriatic arthritis across six studies [1]. There has been specific interest in the co-occurrence of fibromyalgia and axSpA for two reasons. The first is a result of a United States Food and Drug Administration Arthritis Advisory Committee meeting in 2013 which considered the case for expanding the use of Tumour Necrosis Factor inhibition (TNFi) therapy from ankylosing spondylitis to non-radiographic axSpA. The application was not approved partly because of concerns about the inappropriate use of such therapy for conditions such as back pain and fibromyalgia in the presence of minor magnetic resonance imaging (MRI) changes or positive HLA-B27 results [2]. The second reason is around understanding the mechanisms of development of fibromyalgia. One hypothesis is that peripheral nociception, if sustained such as in axSpA, could in the context of an individual susceptible to its development, lead to central sensitisation and the development of fibromyalgia . An alternative possibility is that high levels of disease activity, and consequent pain, poor function and impact on quality of life including work, lead to emotional distress which itself has been shown to increase the risk of fibromyalgia. [3].

The British Society for Rheumatology Biologics Register (BSRBR-AS) of patients with axSpA is by far the largest study to have examined fibromyalgia as a comorbidity in this condition. In analysis of 1504 patients, it reported that 20.7% met the 2011 research criteria for fibromyalgia [4, 5]. Those with co-morbid fibromyalgia had high levels of unmet need; this included substantially worse disease activity scores, function, global status (all measured using Bath indices) and quality of life [4], findings which have been consistent across studies [6, 7]. If persons with poorly controlled disease are more likely to fulfill criteria for fibromyalgia through the process of central sensitisation, then management should focus on reducing disease activity associated with axSpA. Alternatively if the co-morbid fibromyalgia-like symptoms are unrelated to disease activity and arise through distinct mechanisms, then management should focus on the fibromyalgia (in addition to any management necessary for axSpA).

In this analysis, using BSRBR-AS, we aimed to establish if distinct clusters of patients with axSpA exist, and if so to a) ascertain whether such clusters exhibit important differences in the prevalence of fibromyalgia and b) determine features of the clusters which exhibit a high prevalence of fibromyalgia.

## Methods

BSRBR-AS is a prospective cohort study which recruited biologic-therapy naïve patients from across Great Britain fulfilling Assessment of SpondyloaArthritis international Society (ASAS) criteria for axSpA [8]. Recruitment for the study took place between December 2012 and December 2017 across 83 secondary care rheumatology centres. Initially only those fulfilling the imaging ASAS criteria were eligible for inclusion, however from November 2014 those meeting the clinical arm were also eligible. The full protocol has been published previously [9]. Patients were recruited to one of two sub-cohorts: those about to commence a biologic therapy (adalimumab, etanercept or certolizumab pegol) and those continuing on non-biologic therapy. The biologic cohort was followed up at 3 months and 6 months, and both cohorts were followed-up at 12 months and yearly thereafter up to a maximum of 5 years. If a patient in the non-biologic cohort commenced biologic therapy they switched sub-cohort and started a new follow-up schedule.

Clinical data collected during recruitment and follow-up appointments included: the presence of extra-spinal manifestations (history of uveitis, psoriasis, inflammatory bowel disease (IBD), peripheral joint involvement, dactylitis and enthesitis), history of comorbidities and physician-assessed swollen and tender joint count (40 and 44 joints respectively), and Bath metrology index (BASMI). In addition to clinical data, patient reported questionnaires were mailed at the same time and included validated instruments assessing, among others: Bath indices of disease activity (BASDAI), function (BASFI), global assessment (BAS-G), mental health (Hospital Anxiety and Depression Scale (HADs) (anxiety and depression subscales each scored 0–21) [10]), fatigue (Chalder fatigue scale, scored 0–11 [11]) and sleep disturbance (Jenkins Sleep Evaluation Questionnaire, scored 0–20 [12]). From August 2015, the patient reported questionnaire included the 2011 modification of the 2010 ACR criteria for fibromyalgia [5]. As the aim of the current analysis was to identify discrete clusters within the axSpA population, in which the prevalence of fibromyalgia would be calculated; only participants who had completed a questionnaire after August 2015 were eligible for inclusion and amongst those who had, the first completion of the fibromyalgia research criteria was used as the time-point for data included in the current analysis.

Cluster analysis classifies individuals into groups (clusters) which optimise homogeneity within groups and heterogeneity between groups, based on a selection of pre-defined characteristics (clustering variables). The groups formed are highly dependent on the variables offered for clustering, therefore, the choice of these is ideally underpinned by empirical evidence. As the number of clusters is not known prior to analysis, a common approach is to determine the optimal clustering solution in one sample and to validate in a second sample. The choice of variables for the current analysis was determined through simple descriptive statistics (t-tests) in which those factors associated with fibromyalgia at *p* ≤ 0.05 were considered important. To mitigate the effects of any differences in measurement scale used across clustering variables, and to adjust for non-normal distribution; each variable was standardised through z-score transformation. Prior to analysis, the eligible BSRBR-AS population was split into two equal-sized samples in which the optimal clustering solution was developed (Sample A) and then validated (Sample B). A three-stage approach was chosen:

- *Stage 1 -* An agglomerative hierarchical cluster analysis was applied to Sample A using the Euclidean distance measure and weighted-average linkage method. The optimal number of cluster solutions was determined through consultation of the dendrogram and agglomeration schedule.
- *Stage 2 -* The optimal solution from stage 1 was validated in Sample B using K-means clustering. The characteristics of each cluster was assessed and compared against those identified by the hierarchical analysis. Where the clustering solutions appeared identical, or near-identical, the solution was considered validated.
- *Stage 3 -* Once the optimal solution was determined and validated (stages 1 & 2) the K-means clustering was conducted once more within Samples A and B combined to identify the final groupings of all participants. These clusters were examined in terms of both the clustering variables used (mean and standard deviations of non-transformed values) and the prevalence of fibromyalgia (or more specifically meeting research criteria for fibromyalgia).

On completion of the clustering procedure, the final clusters were examined to explore differences in both clinician and patient-reported factors. Demographic characteristics included: age, age at symptom onset, gender, smoking and alcohol use, while clinical factors included: classification criteria met, treatments prescribed and spinal mobility (BASMI: scored 0 (least) - 10 (most) severe [13]). Patient reported measures of health, from questionnaires, included the BASDAI, BASFI and BAS-G: all scored 0 (least) - 10 (most) severe [14–16]) and spinal pain (scored 0 (least) - 10 (most) severe). Quality of life was assessed by the Ankylosing Spondylitis Quality of Life Index (ASQoL: scored 0 (good) to 18 (poor) [17]) and the short form 12 (scored 0 (poor) to 100 (best) [18]). Participants were asked to report co-morbidities including: myocardial infarction, unstable angina, congestive heart failure, stroke, hypertension, diabetes, asthma, chronic bronchitis/emphysema, peptic ulcer, liver disease, renal disease, tuberculosis, demyelination, depression and cancer. This allowed a co-morbidity “count” to be derived. Lastly, employment status was assessed by the Work Productivity and Activity Impairment scale (WPAI:SHP) to give an indication of work absence (absenteeism), impairment in work-productivity (presenteeism), overall work and non-work activity impairment (all scored as 0–100% [19]). From the information collected, the Ankylosing Spondylitis Disease Activity Scale (ASDAS) was calculated using the measure of CRP (preferentially) or ESR closest to the patient completed questionnaire used, provided it was within 90 days [20]. In addition to calculating the proportion of participants within each cluster meeting criteria for fibromyalgia, the sub-scales of the criteria, namely the Widespread Pain Index (WPI, score 0–19) and Symptom Severity Score (SSS, score 0–12) could be calculated. Differences were assessed using chi-square or t-tests as appropriate and results are given as proportions or means (with 95% Confidence Intervals). To determine if similar clusters exist *within* the subgroup of participants meeting research criteria for fibromyalgia, this subgroup was split into two equal-sized samples (C and D) and the entire clustering process described above was repeated.

All analysis was conducted on the August 2017 dataset using STATA (StataCorp LP version 15.0).

## Results

In total 1338 participants were eligible for the current analysis of whom 65% were male, with a median age of 49 years, and median time since symptom onset of 18 years, and 36% had been recruited to the biologic cohort of the study. Of those tested, 79% were HLA-B27 positive. Most participants (64.6%) met the modified New York (mNY) criteria for ankylosing spondylitis, a further 29.7% fulfilled the ASAS imaging criteria for axSpA but not mNY, while 5.7% only met ASAS clinical criteria for axSpA. At the time when first completing research criteria for fibromyalgia, 23% (*n* = 307) were classified positive. Prior to further analysis, the study population was randomly split in two equal sized groups.

Factors significantly associated with meeting fibromyalgia research criteria were identified and were eligible to be used in the cluster analysis. Where an eligible variable was strongly related to another eligible variable, only the factor with the strongest relationship to fibromyalgia was used for clustering. The final variable group used for clustering was: number of extra-spinal manifestation and co-morbidity count, swollen joint count, tender joint count, anxiety, depression, fatigue and sleep disturbance.

**Fig. 1:**
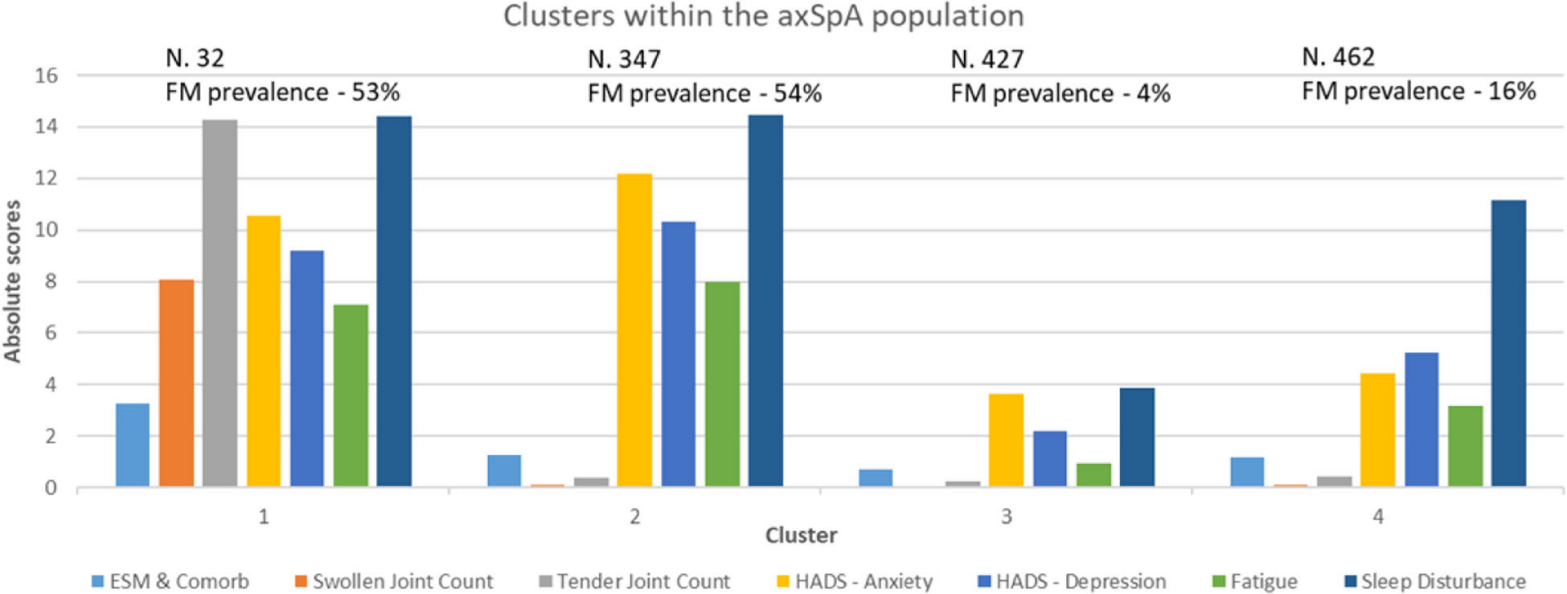
Cluster solutions within whole BSRBR-AS population

The results of the hierarchical analysis in Sample A indicated the presence of 4 distinct clusters which were validated in Sample B with the K-means analyses. Differences in the clustering factors across each of the 4 clusters for samples A and B combined are detailed in Table 1 and Fig. 1. There was one small cluster (Cluster 1) with 32 subjects. It was characterised by high scores or levels across all clustering variables and amongst participants in this cluster there was a very high proportion of participants who met research criteria for fibromyalgia (53%). The remaining clusters were of roughly equal size (varying between 347 and 462 subjects). Cluster 2 was characterised by few extra-spinal manifestations and comorbidities, low number of tender and swollen joints but high levels of anxiety, depression, fatigue and sleep disturbance. This cluster also had a very high proportion meeting research criteria for fibromyalgia (54%). Participants classified in Cluster 3 had few extra-spinal manifestations or comorbidities, a low number of tender and swollen joints low levels of anxiety, depression, fatigue and sleep disturbance. There was a low proportion meeting research criteria for fibromyalgia (4%). Finally Cluster 4 was characterised by few extra-spinal manifestations or comorbidities, a low number of tender and swollen joints, low levels of anxiety, depression and fatigue, but moderate sleep disturbance. There was a moderate proportion meeting research criteria for fibromyalgia (16%).

**Table 1:** Clustering variables across clusters (total population) and proportion meeting research criteria for fibromyalgia

N	Cluster 1	Cluster 2	Cluster 3	Cluster 4
	32	347	427	462
	Mean (95% CI)	Mean (95% CI)	Mean (95% CI)	Mean (95% CI)
Clustering Factors				
Extra-spinal manifestation & comorbidity count	3.3 (2.7, 3.9)	1.3 (1.1, 1.4)	0.7 (0.6, 0.8)	1.2 (1.1,1.3)
Swollen joint count	8.1 (5.5, 10.7)	0.1 (0.03, 0.2)	0.05 (0.01, 0.1)	0.1 (0.05, 0.13)
Tender joint count	14.3 (11.2, 17.3)	0.4 (0.2, 0.5)	0.2 (0.1, 0.3)	0.4 (0.3, 0.6)
Anxiety score	10.5 (8.6, 12.5)	12.2 (11.8, 12.5)	3.6 (3.4, 3.9)	4.5 (4.2, 4.7)
Depression score	9.2 (7.6, 10.8)	10.3 (10.0, 10.7)	2.2 (2.0, 2.4)	5.2 (5.0, 5.5)
Fatigue score	7.1 (5.9, 8.3)	8.0 (7.7, 8.3)	0.9 (0.8, 1.1)	3.2 (2.9,3.4)
Sleep disturbance score	14.4 (12.4, 16.5)	14.5 (14.0, 15.0)	3.9 (3.6, 4.2)	11.2 (10.7, 11.6)
FM research criteria (and components)				
Proportion positive (%)	53%	54%	4%	16%
Widespread pain index	7.2 (6.0, 8.5)	7.4 (7.0, 7.8)	2.9 (2.7, 3.2)	5.0 (4.7, 5.3)
Symptom severity score	7.9 (6.9, 8.9)	8.4 (8.2, 8.7)	2.7 (2.5, 2.9)	5.4 (5.2, 5.6)

Examining factors which were not used in the clustering (Table 2), it is notable that the members of Clusters 1 and 2, with more than half meeting criteria for fibromyalgia, had markedly worse axSpA disease activity, function, global status, spinal pain, poorer mental and physical health. Both clusters had mean ASDAS values in the “very high disease activity” range. (i.e. > 3.5). Quality of Life and work impact were also worst in Clusters 1 and 2, with intermediate levels in Cluster 4 in comparison to Cluster 3. Clusters 1 and 2 were the most likely to be receiving biologic therapy (31 and 39% respectively) followed by Cluster 4 (24%) and Cluster 3 (14%). There were approximately double the proportion of smokers in Clusters 1 and 2 (25 and 29% respectively) compared to Clusters 3 and 4 (13 and 14%), however in contrast, more had given up alcohol (28 and 28% v. 10 and 14%). Cluster 1 was distinguished by having a much higher proportion of female members (59%) than any other cluster (30–40%).

**Table 2:** Differences in clinical and patient reported characteristics (not used in clustering) across clusters (total population)

	Cluster 1	Cluster 2	Cluster 3	Cluster 4	*p* value
	N (%)	N (%)	N (%)	N (%)	
Categorical variable					
Gender					
male	13 (40.6)	209 (60.2)	297 (69.6)	298 (64.5)	*p* < 0.001
female	19 (59.4)	138 (39.8)	130 (30.4)	164 (35.5)	
Smoking Status					
never	10 (31.2)	129 (37.6)	201 (47.4)	214 (46.8)	*p* < 0.001
ex	14 (43.8)	116 (33.8)	168 (39.6)	180 (39.4)	
current	8 (25.0)	98 (28.6)	55 (13.0)	63 (13.8)	
Alcohol Use					
never	1 (3.1)	39 (11.4)	20 (4.7)	25 (5.5)	*p* < 0.001
ex	9 (28.1)	94 (27.5)	41 (9.7)	63 (13.9)	
current	22 (68.8)	209 (61.1)	361 (85.6)	366 (80.6)	
Employed					
no	15(46.9)	164 (47.3)	113 (26.5)	160 (34.9)*	*p* < 0.001
yes	17 (53.1)	183 (52.7)	314 (73.5)	299 (65.1)	
Job type					
mainly sedentary	8 (50.0)	81 (45.5)	171 (56.1)	179 (61.5)	*p* = 0.009
mainly physical	8 (50.0)	97 (54.5)	134 (43.9)	112 (38.5)	
Current biologic therapy					
no	22 (68.8)	211 (61.0)	364 (86.3)	350 (76.1)*	*p* < 0.001
yes	10 (31.2)	135 (39.0)	58 (13.7)	110 (23.9)	
NSAID (last 6 m)					
no	7 (21.9)	114 (32.9)	136 (31.9)	142 (30.7)	*p* = 0.611
yes	25 (78.1)	233 (67.1)	291 (68.1)	320 (69.3)	
DMARD use in past 6 m					
no	21 (65.6)	305 (87.9)	374 (87.6)	401 (86.8)	*p* = 0.004
yes	11 (34.4)	42 (12.1)	53 (12.4)	61 (13.2)	
HLA B27 status					
positive	20 (62.5)	148 (42.7)	260 (60.9)	242 (52.4)*	*p* < 0.001
negative	7 (21.9)	58 (16.7)	52 (12.2)	66 (14.3)	
untested	5 (15.6)	141 (40.6)	115 (26.9)	154 (33.3)	
	Mean (95% CI)	Mean (95% CI)	Mean (95% CI)	Mean (95% CI)	
Age					
years	49.5 (45.2, 53.8)	47.5 (46.0, 48.9)	49.4 (48.1, 50.7)	50.3 (48.9, 51.6)	*p* = 0.062
Age at symptom onset					
years	31.7 (27.7, 35.7)	30.7 (29.3, 32.0)	28.8 (27.8, 29.8)	29.7 (28.6, 30.8)	*p* = 0.074
Disease Activity					
BASDAI: 0 (best) – 10 (worst)	6.7 (5.9, 7.4)	6.7 (6.5, 6.9)	2.5 (2.3, 2.6)	4.5 (4.3, 4.7)*	*p* < 0.001
Disease Activity					
ASDAS Score	3.6 (3.2, 4.0)	3.7 (3.6, 3.8)	2.2 (2.1, 2.3)	2.9 (2.7, 3.0)*	*p* < 0.001
Physical Function					
BASFI: 0 (best) – 10 (worst)	6.2 (5.4, 6.9)	6.5 (6.3, 6.8)	2.5 (2.3, 2.7)	4.3 (4.0, 4.5)*	*p* < 0.001
Spinal Mobility					
BASMI: 0 (best) – 10 (worst)	4.2 (3.6, 4.8)	4.1 (3.8, 4.3)	3.4 (3.2, 3.6)	3.7 (3.4, 3.9)	*p* < 0.001
Patient Global					
BASG: 0 (best) – 10 (worst)	6.9 (6.2, 7.5)	7.0 (6.8, 7.2)	2.6 (2.4, 2.8)	4.6 (4.3, 4.8)*	*p* < 0.001
Spinal Pain					
VAS: 0 (best) – 10 (worst)	5.1 (4.1, 6.2)	6.3 (6.1, 6.6)	2.2 (2.0, 2.4)	4.0 (3.7, 4.2)*	*p* < 0.001
SF12 Mental Component					
100 (best) – 0 (worst)	37.6 (33.0, 42.1)	35.2 (34.3, 36.1)	54.0 (53.3, 54.6)	47.2 (46.3, 48.0)*	*p* < 0.001
SF12 Physical Component					
100 (best) – 0 (worst)	32.3 (29.3, 35.3)	32.3 (31.2, 33.4)	46.2 (45.3, 47.1)	39.4 (38.4, 40.5)*	*p* < 0.001
Quality of Life					
ASQoL: 0 (best) – 18 (worst))	12.9 (11.3, 14.4)	13.3 (12.9, 13.7)	3.0 (2.7, 3.3)	7.9 (7.5, 8.3)*	*p* < 0.001
Work absence					
absenteeism (%)	17.4 (0.1, 34.7)	11.2 (7.7, 14.6)	0.4 (0.1, 0.7)	4.4 (2.6, 6.1)*	*p* < 0.001
Work impairment					
presenteeism (%)	48.6 (36.4, 60.7)	52.5 (49.0, 56.1)	15.1 (13.1, 17.0)	28.8 (26.3, 31.4)*	*p* < 0.001
Overall work impairment					
(%)	48.8 (35.7, 62.0)	55.1 (51.3, 58.9)	15.4 (13.4, 17.5)	30.4 (27.7, 33.2)*	*p* < 0.001
Other activity impairment					
(%)	63.1 (53.8, 72.4)	65.4 (63.1, 67.6)	19.8 (17.9, 21.7)	38.4 (36.1, 40.6)*	*p* < 0.001

*significant difference between cluster 3 & 4 at *p* < 0.05

Participants meeting research criteria for fibromyalgia were split into two samples (C and D). The results of the hierarchical analysis on Sample C indicated that there were three distinct clusters which was validated in the K-means analysis using Sample D. The 3 cluster solution using both Samples C and D combined is shown in Table 3. Cluster 1 was small (*n* = 17) with members scoring very highly on tender and swollen joints, anxiety, depression, fatigue and sleep problems and consequently had high pain and symptom severity scores on the fibromyalgia research criteria. This cluster was predominantly female (77%), in contrast to the other clusters which had 40–48% female members. Cluster 2 was larger (*n* = 157), with average characteristics very similar to Cluster 1 except that almost all members had no swollen or tender joint and had lower levels of co-morbidities and extra-spinal manifestations. Nevertheless the WPI and SSS were very similar between Clusters 1 and 2. In contrast, subjects in Cluster 3 (*n* = 120) scored lower across all domains and consequently had average WPI scores lower by between 1.3–1.5 and SSS lower by between 2.0–2.2.

**Table 3:** Clustering variables across clusters and fibromyalgia criteria sub-scale scores (amongst participants who met criteria for fibromyalgia)

N	Cluster 1	Cluster 2	Cluster 3
	17	157	120
	Mean (95% CI)	Mean (95% CI)	Mean (95% CI)
Clustering Factors			
Extra-spinal manifestation & comorbidity count	3.7 (2.8, 4.6)	1.5 (1.2, 1.7)	1.3 (1.0, 1.5)
Swollen joint count	7.1 (4.2, 10.0)	0.08 (0.002, 0.16)	0.1 (0.01, 0.13)
Tender joint count	15.0 (11.7, 18.3)	0.5 (0.2, 0.8)	0.4 (0.1, 0.7)
Anxiety *(HADs - scored 0–21)*	12.5 (9.8, 15.1)	13.2 (12.6, 13.7)	8.1 (7.5, 8.7)
Depression *(HADs - scored 0–21)*	10.8 (8.7, 12.9)	11.2 (10.7, 11.7)	6.2 (5.7, 6.7)
Fatigue *(Chalder Fatigue - scored 0–11)*	8.9 (7.6, 10.2)	8.5 (8.1, 8.9)	4.6 (4.0, 5.2)
Sleep disturbance *(Jenkins - scored 0–20)*	18.1 (16.6, 19.6)	16.0 (15.4, 16.6)	10.1 (9.1, 11.1)
FM components			
Widespread pain index	9.4 (7.7, 11.0)	9.2 (8.6, 9.8)	7.9 (7.3, 8.4)
Symptom severity score	9.5 (8.6, 10.4)	9.7 (9.4, 10.0)	7.5 (7.2, 7.9)

Examining factors which were not used in the clustering of fibromyalgia patients (Table 4) Clusters 1 and 2 were very similar with respect to almost all the characteristics examined although Cluster 1 had primarily female members and members who were less likely to have recent use of DMARDs. Cluster 3 had better disease activity, although all three fibromyalgia patient clusters had ASDAS scores in the “very high disease activity” range. Cluster 3 also had better function, physical and particularly mental health, quality of life and work parameters.

**Table 4:** Differences in clinical and patient reported characteristics (not used in clustering) across clusters (fibromyalgia positive participants)

	Cluster 1	Cluster 2	Cluster 3	
	N (%)	N (%)	N (%)	*p* value
Gender				
male	4 (23.5)	94 (59.9)^a^	63 (52.5)	*p* = 0.014
female	13 (76.5)	63 (40.1)	57 (47.5)	
Smoking Status				
never	5 (29.4)	53 (34.2)	51 (43.6)	*p* = 0.027
ex	6 (35.3)	52 (33.5)	48 (41.0)	
current	6 (35.3)	50 (32.3)	18 (15.4)	
Alcohol Use				
never	1 (5.9)	21 (13.5)	13 (11.1)	*p* = 0.032
ex	7 (41.2)	50 (32.3)	21 (18.0)	
current	9 (52.9)	84 (54.2)	83 (70.9)	
Employed				
no	8 (47.1)	85 (54.1)	47 (39.5)	*p* = 0.054
yes	9 (52.9)	72 (45.9)	72 (60.5)	
Job type				
mainly sedentary	1 (12.5)	30 (43.5)	39 (55.7)	*p* = 0.044
mainly physical	7 (87.5)	39 (56.5)	31 (44.3)	
Current biologic therapy				
no	13 (76.5)	87 (55.4)	74 (61.7)	*p* = 0.189
yes	4 (23.5)	70 (44.6)	46 (38.3)	
NSAID (last 6 m)				
no	3 (17.6)	55 (35.0)	32 (26.7)	*p* = 0.160
yes	14 (82.4)	102 (65.0)	88 (73.3)	
DMARD use in past 6 m				
no	10 (58.8)	136 (86.6)^a^	102 (85.0)	*p* = 0.011
yes	7 (41.2)	21 (13.4)	18 (15.0)	
	Mean 95% CI	Mean (95% CI)	Mean (95% CI)	
Age				
years	49.2 (43.3, 55.0)	47.8 (45.7, 50.0)	50.4 (47.8, 53.0)	*p* = 0.445
Age at symptom onset				
years	29.3 (25.6, 33.0)	29.7 (27.7, 31.7)	29.7 (27.5, 31.9)	*p* = 0.873
Disease Activity				
BASDAI: 0 (best) – 10 (worst)	7.8 (7.2, 8.5)	7.4 (7.2, 7.6)	6.0 (5.7, 6.2)	*p* < 0.001
Disease Activity				
ASDAS	3.8 (3.4, 4.2)	3.9 (3.8, 4.1)	3.3 (3.2, 3.5)	*p* < 0.001
Physical Function				
BASFI: 0 (best) – 10 (worst)	7.2 (6.3, 8.1)	7.2 (6.9, 7.5)	5.5 (5.1, 5.9)	*p* < 0.001
Spinal Mobility				
BASMI: 0 (best) – 10 (worst)	4.3 (3.4, 5.1)	4.2 (3.8, 4.6)	4.1 (3.6, 4.6)	*p* = 0.934
Patient Global				
BASG: 0 (best) – 10 (worst)	7.7 (7.1, 8.3)	7.7 (7.4, 7.9)	6.1 (5.7, 6.5)	*p* < 0.001
Spinal Pain				
0 (best) – 10 (worst)	6.2 (4.7, 7.6)	7.1 (6.8, 7.4)	5.6 (5.1, 6.0)	*p* < 0.001
SF12 Mental Component				
100 (best) – 0 (worst)	31.4 (26.6, 36.3)	32.5 (31.1, 34.0)	44.2 (42.5, 45.9)	*p* < 0.001
SF12 Physical Component				
100 (best) – 0 (worst)	28.9 (25.0, 32.8)	29.9 (28.4, 31.5)	34.3 (32.4, 36.2)	*p* < 0.001
Quality of Life				
ASQoL: 0 (best) – 18 (worst)	15.4 (14.2, 16.7)	14.6 (14.1, 15.1)	10.9 (10.2, 11.5)	*p* < 0.001
Work absence				
absenteeism: %	7.0 (1.3, 12.7)	14.2 (8.2, 20.1)	9.3 (4.4, 14.1)	*p* = 0.737
Work impairment				
presenteeism: %	55.6 (42.4, 68.7)	60.3 (55.1, 65.6)	44.8 (39.8, 49.8)	*p* < 0.001
Overall work impairment				
%	56.4 (41.3, 71.6)	63.4 (58.0, 68.9)	48.0 (42.7, 53.3)	*p* < 0.001
Other activity impairment				
**%**	75.9 (66.4, 85.4)	72.0 (69.4, 74.7)	54.5 (50.2, 58.8)	*p* < 0.001

^a^significant difference between cluster 1 & 2 at *p* < 0.05

## Discussion

We have found evidence of distinct groups of axSpA patients: those with high disease activity which is either mainly axial or (in a smaller group) both axial and peripheral and in whom more than half of persons meet criteria for fibromyalgia; patients with low disease activity (in whom the prevalence of fibromyalgia is similar to persons without axial spondyloarthritis); and a group of patients with intermediate disease activity but with high levels of sleep disturbance and a raised prevalence of fibromyalgia. Within patients who meet criteria for fibromyalgia, there are two groups with higher axSpA disease activity (one with primarily axial disease and a smaller group with axial and peripheral disease) and this is reflected in higher pain and symptom severity scores of the fibromyalgia research criteria, in comparison to a third group.

The strength of this study was that it used a large national register to which most patients with axial spondyloarthritis were eligible to be enrolled. In examining clusters it used a split sample approach for their development and validation. It found consistent results – there were similar clusters within the total axSpA participant group and the sub-group who met research criteria for fibromyalgia. The clusters within the population group exhibited proportions meeting the research criteria for fibromyalgia which varied from the norm in the general population (~ 2–5%) ( [21] to two groups with a prevalence of more than 50%. There are some methodological issues to be considered in the interpretation. Ideally the cluster structure should be confirmed in an external dataset. Not all patients with axSpA meeting ASAS criteria were eligible to join the register – those patients who had already commenced biologic therapy or had previous experience of biologic therapy were not eligible to be enrolled. The overall proportion of biologic therapy patients recruited was 7% lower than the proportion reporting taking biologic therapy in a recent survey of 1979 members of the National Ankylosing Spondylitis Society – the UK patient support group (36% v. 43%) [22]. The relative size of the clusters should be considered indicative, therefore. This is particularly true with respect to patients who meet only the clinical arm of the ASAS criteria. They were only eligible for the registry in the latter 3 years of the 5-year recruitment period. We therefore examined the relative sizes of the clusters if only this latter period was considered. For all patients the distribution (for 1000 nominal patients) changed from 25:274:337:364 across Clusters 1–4 to 25:296:302:377 and for FM patients from 58:534:408 across Clusters 1–3 to 62:541:397. Thus it can be seen that the relatively sizes of the clusters are changed very little when we consider only the period over which patients meeting the clinical criteria of ASAS were eligible.

The second methodological issue is that the patient data used in this study varied with respect to their entry into the study. Some patients who were enrolled later in the recruitment period would have completed the fibromyalgia criteria at baseline or at one of the first follow-ups while for those recruited early it may have been up to 2.5 years before they completed their fibromyalgia assessment. Thus for the biologic therapy group, they will have completed this at various points in their history of such therapy. Finally the 2011 research criteria for fibromyalgia have not specifically been validated in the context of inflammatory arthritis. Indeed the criteria as published exclude persons if their pain could be explained by another condition. However almost all studies which have implemented the 2011 research criteria have dropped this question as it is considered difficult to evaluate and indeed it has been removed from the 2016 revision of the criteria [23]. We note however that in the cluster analysis of all axSpA patients, most of the axSpA patients with high swollen and tender joint count were in Cluster 1, and that cluster has a very high prevalence of fibromyalgia. It is possible that such peripheral involvement may result in high numbers of body regions scored as painful in the fibromyalgia criteria (although the influence on abdominal pain and headache aspects of the criteria is less obvious).

The results of the current study show that inflammation is strongly associated with meeting criteria for fibromyalgia. The clusters with high disease activity all had a high prevalence of fibromyalgia. Basu et al. [24] have shown that RA patients who have features of fibromyalgia (what they call “fibromyalgianess”), demonstrate similar neurobiologic features, on imaging, to that observed in fibromyalgia patients. A further study reported that high levels of inflammation in RA were associated, on MRI, with more positive connections between the inferior parietal lobule, medial prefrontal cortex, and multiple brain networks, as well as reduced inferior parietal lobule grey matter, and that these patterns of connectivity were associated with reported fatigue, pain and cognitive dysfunction [25]. The authors postulate that such networks may provide a mechanism by which peripheral inflammation results in central changes and features typically associated with fibromyalgia, although to what extent this association is mediated through emotional distress remains to be established. When treated with TNFi therapy, axSpA patients in BSRBR-AS with co-morbid fibromyalgia showed a similar absolute improvement in disease activity and quality of life over 6 months compared to those without co-morbid fibromyalgia, and two-thirds no longer satisfied fibromyalgia criteria suggesting that targeting inflammation is important to reduce fibromyalgia symptoms in patients with active axSpA [26].

An alternative explanation is that having fibromyalgia distorts the measures used to assess axSpA. Indeed, Alluno et al. [27] demonstrated that measures thought to be disease specific such as the Bath indices are not axSpA specific. However it is unlikely that this can entirely account for the current observations. Duffield et al. [1] in their meta-analysis of chronic inflammatory arthritis reported that across studies included, patients with axSpA and fibromyalgia had BASDAI scores that were around two points higher than those with axSpA alone (mean difference 2.2 95% CI (1.9, 2.6)). The differences observed in BASDAI between clusters in our study greatly exceed such levels. A previous paper from the BSRBR-AS demonstrated that the presence of co-morbid fibromyalgia increased BASDAI scores, on average only by 1.04 (after adjustment for other features of the disease) and increased the the ASQoL score (indicating poorer quality of life) by 1.42 [26].

However around one-third of patients with axSpA and fibromyalgia still have co-morbid fibromyalgia even after TNFi and those least likely to respond have high scores on the fibromyalgia symptom severity scale [26]. The retention rate on TNFi at 2 years is also lower for axSpA patients with co-morbid fibromyalgia (28% v. 42%) [6]. It seems therefore that even if inflammation is the primary driver of fibromyalgia symptoms, then once developed, therapeutic targeting of inflammatory pathways while important, is not sufficient. Further we have observed in the cluster results of all axSpA, a group of patients with modest disease activity and high levels of sleep disturbance who show a high prevalence of fibromyalgia. Whether additionally using non pharmacologic therapies (such as cognitive behaviour therapies) improves outcomes in such patient groups is not known but evidence in relation to pain (including fibromyalgia) and sleep disorders is promising [28, 29] and is currently being evaluated in ongoing studies of patients with axSpA and fibromyalgia.

## Conclusions

In summary, this analysis has demonstrated distinct groups of axSpA patients with very different likelihood of reporting co-morbid fibromyalgia. The major feature defining clusters with a high prevalence of fibromyalgia is high disease activity and taken together with evidence from previous studies in this population, and others, managing the co-morbid fibromyalgia may be most successful with pharmacologic therapy to target inflammation but enhanced by the concurrent use of non-pharmacologic therapy. This hypothesis awaits testing in formal studies. However the recording of information on features of fibromyalgia is not routine in most clinics assessing axSpA – and it would be important, if we seek to provide appropriate approaches to management to firstly ensure we are collecting relevant information to identify such disease features.

## Abbreviations

ACR: American college of rheumatology
ASAS: Assessment of spondyloarthritis international society
ASDAS: Ankylosing spondylitis disease activity scale
ASQoL: Ankylosing spondylitis quality of life index
axSpA: Axial spondyloarthritis
BASDAI: Bath ankylosing spondylitis disease activity index
BASFI: Bath ankylosing spondylitis functional index
BAS-G: Bath ankylosing spondylitis global score
BASMI: Bath ankylosing spondylitis metrology index
BSRBR:AS: British Society for Rheumatology biologics register
CI: Confidence interval
DMARD: Disease-modifying antirheumatic drug
HADs: Hospital anxiety and depression scale
IBD: Inflammatory bowel disease
mNY: Modified New York
MRI: Magnetic resonance imaging
RA: Rheumatoid arthritis
SSS: Symptom severity score
TNFi: Tumour necrosis factor inhibition
WPAI:SHP: Work productivity and activity impairment scale: specific health problem
WPI: Widespread pain index
